# Essential ICU drug shortages for COVID-19: what can frontline clinicians do?

**DOI:** 10.1186/s13054-020-02971-x

**Published:** 2020-05-26

**Authors:** Wen Ting Siow, Simeon H. Tang, Rohit Vijay Agrawal, Addy Y. H. Tan, Kay Choong See

**Affiliations:** 1 Division of Respiratory and Critical Care Medicine, Department of Medicine, National University Hospital, National University Health System, 1E Kent Ridge Rd, Singapore, 119228 Singapore; 2 Department of Pharmacy, National University Hospital, Singapore, Singapore; 3 Department of Anaesthesia, National University Hospital, Singapore, Singapore

Dear Editor,

As the coronavirus disease 2019 (COVID-19) pandemic persists, preparing intensive care units (ICUs) for sustained service becomes a challenge. Based on China’s experience, Li and colleagues highlighted ten critical issues [1], top-most being a severe shortage of critical medical resources including physicians, nurses, and ICU beds. We would like to highlight a related issue in Singapore that will likely also apply elsewhere. Despite having adequate staff, beds, and equipment, supply chain disruptions has led to several ICU drugs being in short supply. For instance, drugs like propofol, atracurium, and noradrenaline have been projected to last less than 1 month in Singapore without fresh supplies.

From an organizational standpoint, the American Society of Health-System Pharmacists has provided valuable guidance regarding operational assessment, therapeutic assessment, shortage impact analysis, and inventory system changes [2]. Adding to a systems approach, frontline clinicians can help alleviate these drug shortages by identifying the drugs in short supply, considering alternatives and assessing the risks when using these alternatives (Table 1).

**Table 1:** Essential ICU drugs and suggestions to manage drug shortages

Preferred drug	Alternatives to first-line agents	Clinical considerations and contraindications
**Analgesics**		
**Fentanyl (IV)**	**Non-opioid analgesics (Enteral/IV)** e.g., acetaminophen and nonsteroidal anti-inflammatory drugs	• Can be used as part of analgesic ladder, barring conventional contraindications
	**Morphine (IV)** • As infusions and/or breakthrough boluses	• Avoid in patients with renal and hepatic impairment • Associated with higher rates of ICU delirium, especially in elderly • May cause histamine release
	**Ketamine (IV)** • As infusion in mechanically ventilated patients	• Unlabeled use as an adjunct to opioid analgesia and sedation • To be used together with a benzodiazepine to reduce dissociative effects and agitation • Avoid in patients with tachyarrhythmias, significant hypertension, ischemic heart disease, traumatic brain injury, raised intracranial pressure, prolonged sepsis, hepatic and renal impairment, thyroid storm
	**Remifentanil (IV)** • As infusion in mechanically ventilated patients	• Preferred in hepatic and renal impairment • Rapid onset and offset • No drug interaction concerns with cytochrome P450 isoenzymes
	**Oxycodone (oral/IV)**	• Enteral formulation has good bioavailability and can be used to transition from continuous opioids • Use with caution in patients with renal and hepatic impairment • In patients who are able to swallow, the sustained released coupled with an antagonist formulation provides sustained analgesia with less gastrointestinal side effects and decreased likelihood for abuse
**Propofol (IV)**	**Midazolam (IV)** • Infusion and/or breakthrough boluses	• Useful for deep sedation • Preferred for younger patients (lower risk of delirium) • Less hemodynamic side effects compared to propofol or dexmedetomidine • Avoid in patients with renal or hepatic impairment
	**Dexmedetomidine (IV)** • Infusion for light sedation	• Useful for light sedation and patients who may be extubated soon • May cause bradyarrhythmias, especially when used with fentanyl or beta-blockers to treat hypertension • Can be used to treat alcohol, benzodiazepine and opioid withdrawal. When stopped, rebound hypertension can occur. Treatment with beta-blockers can make rebound hypertension worse due to upregulation of alpha-adrenergic receptors • Cannot be used for patients requiring paralysis
	**Thiopentone (IV)**	• Useful for treatment of status epilepticus and patients with raised intracranial pressure • To use with caution in patients with hemodynamic instability, asthma and hepatic failure
	**Clonidine (oral)**	• Can be used to transit from dexmedetomidine for ICU sedation • Can be used as adjunct to treat opioid withdrawal • To use with caution in patients with hemodynamic instability • Requires gradual weaning in prolonged use
**Neuromuscular blockade**		
**Atracurium (IV)**	**Rocuronium (IV)**	• Some patients may experience prolonged recovery of neuromuscular function especially after prolonged use, in the presence of hepatic and renal impairment or when used with corticosteroids • Minimal histamine release
	**Cisatracurium (IV)**	• Preferred in hepatic and renal impairment • Less accumulation than atracurium after prolonged use • Minimal histamine release
	**Pancuronium (IV)**	• A longer acting neuromuscular blocking agent as an alternative for atracurium, especially in patients who require prolonged paralysis • Can be given as intermittent boluses • Some patients may experience prolonged recovery of neuromuscular function especially after prolonged use, in the presence of hepatic and renal impairment or when used with corticosteroids • Minimal histamine release
**Vasopressors**		
**Noradrenaline (IV)**	**Adrenaline (IV)**	• May precipitate peripheral ischemia, gut ischemia, and lactic acidosis • May cause hyperglycemia
	**Phenylephrine (IV)**	• May precipitate reflex bradycardia and visceral vasoconstriction • May have tachyphylaxis and ceiling effect
	**Dopamine (IV)**	• May precipitate tachyarrythmias. Avoid in uncorrected, pre-existing tachyarrhythmias or malignant tachyarrhythmias, e.g., ventricular fibrillation • Avoid as first-line agent or sole agent for sepsis
**Vasopressin (IV)**	**Terlipressin (IV)**	• Increased risk for digital ischemia with terlipressin infusion
**Others (fluids and medications)**		
**Commonly used solutions include** • Lactated Ringer’s solution • 0.9% sodium chloride (normal saline)	• **Drug dilutions with normal saline can be switched to other compatible solutions:** o Dextrose 5% o Lactated Ringers’ solution o Sterile water o No dilution at all, administered as neat bolus • **Irrigation can be done with alternative solutions:** o Sterile water o Clean/sterilized tap water • **Fluid resuscitation can be done with alternative balanced crystalloid solutions:** o Plasmalyte o Stereofundin	
**Antimicrobials**	• Strong antimicrobial stewardship with daily review of de-escalation or cessation of antimicrobial when clinically appropriate • Select a more frequent dosing regimen for time-dependent antibiotics to optimize pharmacodynamic parameters and minimize wastage • Indicate specific duration of antimicrobials	
**Insulin (short-acting forms)**	• Short-acting insulin is commonly used in ICUs for glycemic control • Requirements per day can be averaged out and converted to a medium to long-acting alternative for glycemic control, accepting slightly more fluctuations in blood glucose levels • Enteral agents can be introduced earlier if the patient has demonstrated clinical stability, to reduce the need for short acting insulin	

*IV* intravenous

Optimizing current drug stocks and reducing waste would require a concerted effort by frontline clinicians. *Physicians* can use light sedation targets or even no sedation with analgesia only, avoid neuromuscular blockade, use train-of-four measurements to avoid overdosing of neuromuscular blockade, and allow permissive hypotension (lowering the mean arterial pressure target to 60–65 mmHg) [3]. *Nurses* can standardize intravenous drug dilutions to negate the need for re-dilution when patients are transferred between different clinical units, use low rather than high concentration drug infusions for more accurate titration to the lowest permissible dose, and perform daily or twice-daily awakening trials for suitable patients. *Pharmacists* can reinforce physician and nursing practice by monitoring drug use patterns, suggesting viable alternatives, checking for drug interactions, and advising on safe administration practices.

It is not inconceivable that even alternative medications can run out, especially in regions that are already resource-limited. In such cases, non-pharmacologic or unconventional measures should be explored. For instance, analgesia may be attained through acupuncture, and anxiety can be alleviated with patient-directed music intervention [4]. Another example is oral midodrine, which is currently being investigated as a means to wean critically ill patients from intravenous vasopressors [5]. Drug shortages may compel clinicians to use oral midodrine as a sole agent for blood pressure augmentation. Physicians, nurses, and pharmacists would then need medicolegal protection when using therapies that are off-label, but that would be necessary for the well-being of patients.

## Data Availability

Data sharing is not applicable to this article as no datasets were generated or analyzed during the current study.
